# Dux Is Dispensable for Skeletal Muscle Regeneration: A Study Inspired by a “Red Flagged” Publication and Editorial Oversight

**DOI:** 10.3390/cells14100695

**Published:** 2025-05-12

**Authors:** Kenric Chen, Erdong Wei, Ana Mitanoska, Micah D. Gearhart, Michael Kyba, Darko Bosnakovski

**Affiliations:** 1 Department of Pediatrics and Lillehei Heart Institute, University of Minnesota, Minneapolis, MN 55455, USA; chen7779@umn.edu (K.C.); ewei@umn.edu (E.W.); mitan001@umn.edu (A.M.); kyba@umn.edu (M.K.); 2 Department of Obstetrics, Gynecology and Women’s Health, University of Minnesota, Minneapolis, MN 55455, USA

**Keywords:** Dux, DUX4, muscle regeneration, Duchenne muscular dystrophy (DMD), mdx, Facioscapulohumeral muscular dystrophy (FSHD)

## Abstract

Double homeobox (DUX) genes are key embryonic regulators that are silenced after the early cleavage stages of embryogenesis. Aberrant expression of DUX4 in skeletal muscle is linked to facioscapulohumeral muscular dystrophy (FSHD). A recent study reported that Dux, the murine ortholog of DUX4, contributes to the dystrophic phenotype in mdx mice, a Duchenne muscular dystrophy (DMD) model, and that its deletion enhances muscle regeneration by reducing oxidative stress. However, convincing evidence of Dux expression in either intact or injured muscle of wild-type (WT) and mdx mice remains lacking, raising questions about its role in muscle homeostasis. To investigate this, we assessed Dux expression in WT and mdx mice and used *Dux* knockout (Dux^Δ/Δ^) mice to evaluate its function during regeneration following cardiotoxin (CTX)-induced injury. Contrary to prior reports, Dux was not expressed in either WT or mdx mice. Moreover, Dux deletion did not enhance muscle regeneration or affect the expression of the oxidative stress regulator Nrf2 following CTX injury. Lastly, we confirmed that neither DUX4 nor its target genes were induced in muscle biopsies from DMD patients, excluding a role for DUX4 in DMD pathology. Collectively, our results demonstrate that Dux does not impact skeletal muscle regeneration or DUX4 contribution to the DMD dystrophic phenotype, directly challenging the conclusions of a previously published study. We comment on issues of editorial oversight that led to the publication of that study and highlight the deleterious impact of the growing wave of fraudulent publications.

## 1. Introduction

The DUX gene family encodes proteins divided into three different clades: DUXA, DUXB, and DUXC [1]. Unlike DUXA and DUXB, the DUXC clade contains a highly conserved C-terminal domain essential for downstream target gene activation and toxicity [2,3]. Mouse Dux (Dux) and human DUX4, members of the DUXC clade, have been shown to activate a set of target genes specific to zygotic genome activation in cleavage-stage embryos [4]. More specifically, Dux and its human ortholog DUX4 are transcribed during the minor wave of zygotic genome activation (ZGA), where they function as transcriptional activators of numerous early embryonic genes [4,5,6,7]. Although *Dux* knockout mouse studies have confirmed its role in ZGA, its function is not essential, as viable offspring can still be produced in its absence [7,8,9]. Furthermore, it has been suggested that Dux may also play a role during implantation development, as some genes involved in placentation are altered [7]. In contrast to in vitro culture, in vivo development of blastocysts is not affected in *Dux* knockout mice; rather, embryos are lost at the post-implantation stages [7].

Under normal conditions, *DUX* genes are epigenetically silenced by DNA methylation [10]. Disruption of this silencing mechanism is linked to facioscapulohumeral muscular dystrophy (FSHD), a genetic myopathy caused by aberrant expression of DUX4 in skeletal muscle cells [11,12,13,14]. High levels of DUX4 expression are toxic to most cell types, including myoblasts [15,16]. Even at low levels, DUX4 impairs myogenic differentiation [15,17]. Similarly, Dux expression is also detrimental, with high levels inducing rapid cell death, while low levels interfere with myogenic pathways [18,19]. Recently, negative cross-regulatory feedback mechanisms among DUX family genes have been reported. For example, Dux has been shown to induce Duxbl expression, promoting its own silencing to facilitate exit from the two-cell stage in embryos [20]. Similarly, DUXA and DUX4c have been found to compete with DUX4, antagonizing its activity and mitigating its muscle toxicity [3,21,22,23].

A recent study by Sun et al. proposed a role for Dux in muscle regeneration and its contribution to the dystrophic phenotype in mdx mice [24]. They reported that Dux deletion alleviated muscle pathology by promoting satellite cell proliferation and reducing oxidative stress by attenuating Nrf2 expression, thereby improving overall survival. Based on these findings, they further speculated that DUX4 might also contribute to Duchenne muscular dystrophy (DMD) pathology [24]. However, these claims are surprising, as no definitive evidence supports Dux expression in murine muscle, nor have DUX4 or its target genes been detected in human muscle outside of individuals with FSHD [25,26,27].

Sabel and colleagues identified several “red flags” for detecting publications reporting work not actually performed. Such publications are often associated with “paper mills” that originate and operate in several developing countries. They estimated that approximately 11% of the scientific output, or about 150,000 papers per year, may be fraudulent in nature [28]. The Sun paper exhibited several of these red flags: the research was reportedly conducted at a clinical institution in China by authors without any prior experience or publications in skeletal muscle biology, the co-authors had no history of collaboration, and all used email addresses not associated with their institutions for correspondence [29,30,31]. Additional concerns included initial claims that the *Dux* knockout mice were obtained from a supplier that did not carry this strain, which was later corrected to a supplier that lists the status of the strain as “developing”. Given the unexpected nature of their findings and the fact that we already had the necessary reagents in hand, we set out to directly test some of their key claims by functionally evaluating the impact of Dux deficiency on muscle regeneration and exploring the potential involvement of DUX4 in DMD.

## 2. Materials and Methods

### 2.1. Mouse Models

C57BL/6J mice (Jackson Laboratories, Bar Harbor, ME, USA) were used as wild-type (WT) controls in all experiments. The *Dux* knockout (Dux^Δ/Δ^) mice were previously generated and characterized in our lab [7]. Mdx mice (C57BL/10ScSn-Dmdmdx/J) were obtained from Jackson Laboratories. The doxycycline (dox)-inducible Dux transgenic mouse model (iDux;HSA) was generated in our lab as previously described (manuscript in preparation) [32]. When treated with dox, either p.o. or i.p., the iDux mice express Dux in skeletal muscle via HSA-rtTA in a similar manner to that of iDUX4;HSA mice, which express DUX4 [32,33]. All animal procedures were conducted in compliance with guidelines and were approved by the University of Minnesota Institutional Animal Care and Use Committee (IACUC, 2206-40184A, approval date 15 September 2022). Mice were maintained under standard housing conditions with free access to food and water, and were monitored regularly for signs of distress.

### 2.2. Cardiotoxin (CTX)-Induced Muscle Injury

To induce muscle injury, 4-week-old Dux^Δ/Δ^ or BL6 control mice were subjected to CTX (cardiotoxin, *Naja pallida*, Sigma-Aldrich, St. Louis, MO, USA) injection. A single 20 μL i.m. injection of a 10 μM CTX solution was administered into the left tibialis anterior (TA) muscle. The right TA muscle served as an uninjured control. Mice were euthanized, and the TA muscles were harvested for analysis at either 2 or 4 weeks post-injury.

### 2.3. Histology

TA muscles were embedded in optimal cutting temperature (OCT) compound and rapidly frozen in liquid nitrogen-cooled isopentane. Ten micrometer tissue sections were cut using a Leica Cryostat and mounted onto glass slides. Hematoxylin and eosin (H&E) staining, as well as Sirius Red/Fast Green staining, were performed on the tissue sections and visualized using an upright Zeiss Axio Observer.Z1 microscope (Zeiss, Oberkochen, Germany). Myofiber cross-sectional analysis and fibrosis quantification were conducted using Cellpose and Fiji ImageJ software. For immunofluorescence staining, tissue samples were fixed in 4% paraformaldehyde (PFA) for 10 min, followed by permeabilization with 0.3% Triton X-100 for 30 min. Sections were incubated overnight at 4 °C in a primary antibody solution containing rabbit anti-Dux [34] and rat anti-laminin (Santa Cruz, Dallas, TX, USA), both diluted 1:1000 in BSA. After three rounds of washing, secondary Alexa Fluor 488 Goat Anti-Rabbit and Alexa Fluor 555 Goat Anti-Rat (Invitrogen, Carlsbad, CA, USA) antibodies were applied at a 1:2000 dilution for 60 min at room temperature. Nuclei were visualized using DAPI staining.

### 2.4. RNA Isolation and RT-qPCR

RNA was extracted using TRIzol (Invitrogen) and an RNA extraction kit (Zymo Research, Tustin, CA, USA). cDNA was synthesized from 0.5 µg of total RNA using an oligo-dT primer and the Verso cDNA Synthesis Kit (Thermo Scientific, Waltham, MA, USA), following the manufacturer’s instructions. Quantitative PCR (qPCR) was performed using SYBR Green (Takara Bio, San Jose, CA, USA). Primer sets used in this study included those for *Col1a1* (F: 5′-GAGCGGAGAGTACTGGATCG and R: 5′-TACTCGAACGGGAATCCATC), *Col3a1* (F: 5′-TGGTCCTCAGGGTGTAAAGG and R: 5′-GTCCAGCATCACCTTTTGGT), *Tgfb* (F: 5′-CTCCCGTGGCTTCTAGTGC and R: 5′-GCCTTAGTTTGGACAGGATCTG), and *Nrf2* (F: 5′-CTTTAGTCAGCGACAGAAGGAC and R: 5′-AGGCATCTTGTTTGGGAATGTG). Gene expression levels were normalized to *Actb* (F: 5′-GGCTGTATTCCCCTCCATCG and R: 5′-CCAGTTGGTAACAATGCCATGT) and analyzed using 7500 System Software (Applied Biosystems, Foster City, CA, USA) with the ∆CT method.

### 2.5. RNA-Seq Data Processing and Visualization

Publicly available RNA-seq data were obtained from the NCBI Sequence Read Archive (SRA) under accession PRJNA391920 and PRJNA976807. Single-end reads were processed using Trim Galore (v0.6.0) with the following parameters: ‘--length 35’, ‘-q 5’, ‘--phred33’, ‘—fastqc’, ‘--stringency 1’, and ‘-e 0.1’. Trimmed reads were aligned to the GRCm38 mouse reference genome using STAR aligner (v2.7.2a) [35]. To accommodate the multicopy nature of the Dux/DUX4 gene, we set ‘—outFilterMultimapNmax’ to ‘100’, allowing reads mapping to up to 100 genomic loci to be retained. BAM files were generated and sorted using Samtools (v1.16.1) [36]. PCR and sequencing duplicates were removed using Picard (v3.3.0) with ‘MarkDuplicates’. For quantification, we used featureCounts from the Rsubread package [37] with Gencode annotations from mouse (M25) or human (V38) using fractional counting (‘fraction = TRUE’) enabled for multi-mapping reads. Reads were treated as unstranded (‘strandSpecific = 0’) and single-end (‘isPairedEnd = FALSE’) or paired (‘isPairedEnd = FALSE’) for the mouse and human data, respectively. To visualize gene expression, we selected the top 50 Dux and DUX4 target genes in myoblasts [4]. These were visualized using the ‘pheatmap’ R package (R 4.3.2 and R 4.4). To ensure a more informative visualization of gene expression without compressing high-expression values, we manually specified non-linear color scale breaks. For Dux, the heatmap color scale was divided into three intervals: 0–1, 1–50, and 50–2500. For DUX4, the color scale was split into two intervals: 0–1 and 1–25. By using explicit breaks instead of a continuous scale, we preserved biological interpretation without visual distortion.

### 2.6. Statistics

Statistical analyses were performed using GraphPad Prism 9.0 software unless otherwise specified. Sample sizes were determined based on prior experience with the assays to ensure sufficient statistical power. Variance was consistent within groups. Differences between groups were assessed using one-way or two-way analysis of variance (ANOVA), followed by Tukey’s post hoc tests. A *p*-value of 0.05 or less was considered statistically significant.

## 3. Results

### 3.1. Lack of Dux Expression in Skeletal Muscle of Mdx Mice and DUX4 in DMD

**Figure 1:**
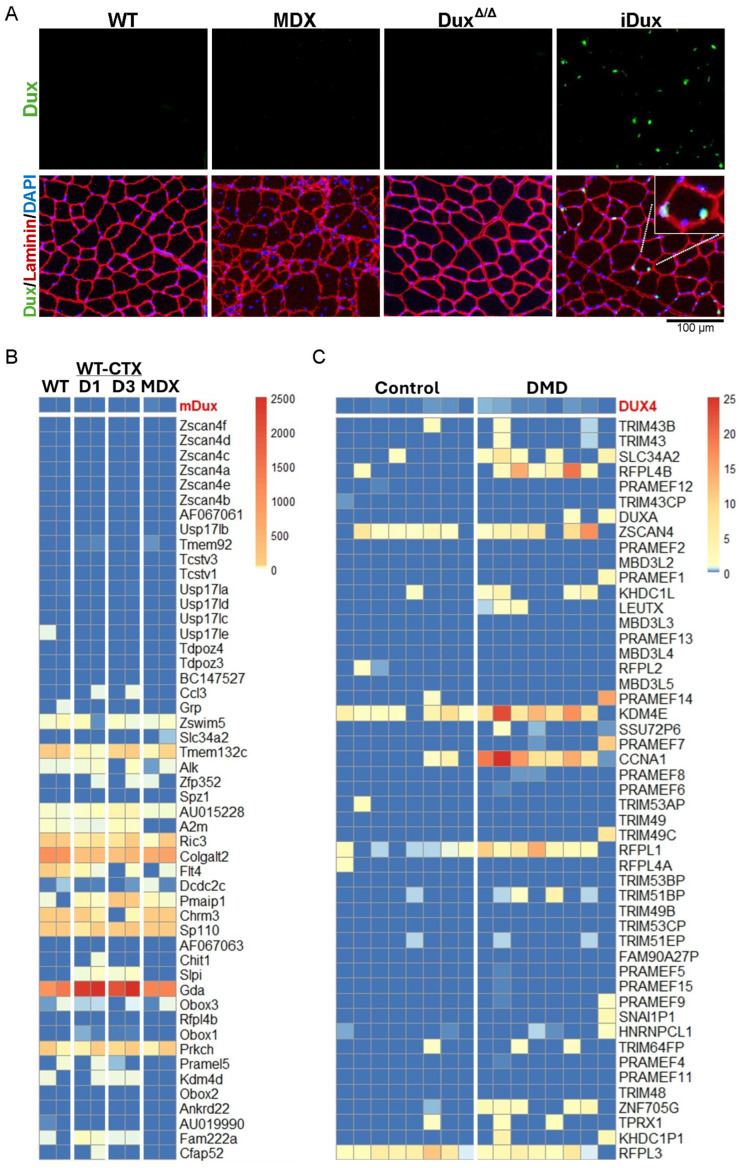
Dux is not expressed in murine skeletal muscle, nor is DUX4 detected in muscle from DMD patients. (**A**) Immunofluorescence staining of tibialis anterior (TA) muscle showing laminin (red), nuclei (DAPI, blue), and Dux (green). iDux mice were induced with a single intraperitoneal injection of doxycycline (100 mg/kg) for 24 h. (**B**) Heatmap showing the expression of *Dux* and the top 50 *Dux* target genes in myoblasts from wild-type (WT), intact, and cardiotoxin (CTX)-injured muscle at 1 and 3 days post-injury, as well as from mdx mice. (**C**) Heatmap showing *DUX4* and the top 50 *DUX4* target gene expression in muscle biopsy samples from Duchenne muscular dystrophy (DMD) patients [40].

Sun et al. reported basal-level expression of *Dux* RNA and protein in the skeletal muscle of wild-type (WT) mice, with a two- to three-fold increase observed in mdx mice, varying by muscle type [24]. This is quite remarkable, given that DUX4 is virtually impossible to detect in human muscle biopsy specimens. When DUX4 protein has been detected in published studies, which examined cultured cells rather than biopsies, it is almost exclusively by immunofluorescence, which can more sensitively detect the very rare nuclei that express it [38,39]. We therefore conducted immunofluorescence staining on tibialis anterior (TA) muscles from WT and mdx mice using a validated anti-Dux antibody [34]. As negative and positive controls, we included TA muscle from *Dux* knockout mice (Dux^Δ/Δ^) and from mice with a doxycycline-inducible *Dux* gene (called iDux;HSA mice) treated with dox (100 mg/kg for 24 h), respectively. Dux protein was undetectable in both WT and mdx muscle samples but was readily detected in the iDux;HSA sample (Figure 1A).

To further confirm the absence of *Dux* expression in skeletal muscle, we analyzed publicly available RNA-seq datasets from both uninjured and cardiotoxin (CTX)-injured muscles of WT and mdx mice [41]. Across all conditions analyzed, *Dux* transcripts were undetectable (Figure 1B). Recognizing that *Dux* expression may be challenging to detect, similar to *DUX4* in FSHD patients [38,39], we next examined the expression profiles of established *Dux* target genes [42,43]. Specifically, we analyzed the top 50 *Dux* target genes previously identified by Whiddon et al. through overexpression studies in murine myoblasts [4]. In line with the lack of *Dux* expression, we observed no induction of *Dux* target genes in mdx muscle compared to WT controls (Figure 1B).

Given that Dux has been proposed to contribute to the muscle phenotype in mdx mice, it has also been hypothesized that DUX4 may play a role in Duchenne muscular dystrophy (DMD). To investigate this possibility, we analyzed transcriptional data from muscle biopsies of DMD patients obtained from two independent studies [40,44]. We assessed both *DUX4* expression and the expression of its top 50 most well-established target genes in skeletal muscle [4,45]. Across all datasets analyzed, we found no detectable *DUX4* transcripts and no significant upregulation of *DUX4* target genes in DMD samples (Figure 1C and Supplementary Figure S1).

Taken together, our data indicates that *Dux* is not expressed in either intact or injured skeletal muscle of wild-type or mdx mice. Furthermore, we found no evidence of *DUX4* expression or activation of its downstream transcriptional program in muscle biopsies from DMD patients. These findings strongly suggest that neither Dux nor DUX4 plays any role in the pathogenesis of DMD.

### 3.2. Dux Does Not Contribute to Muscle Regeneration

**Figure 2:**
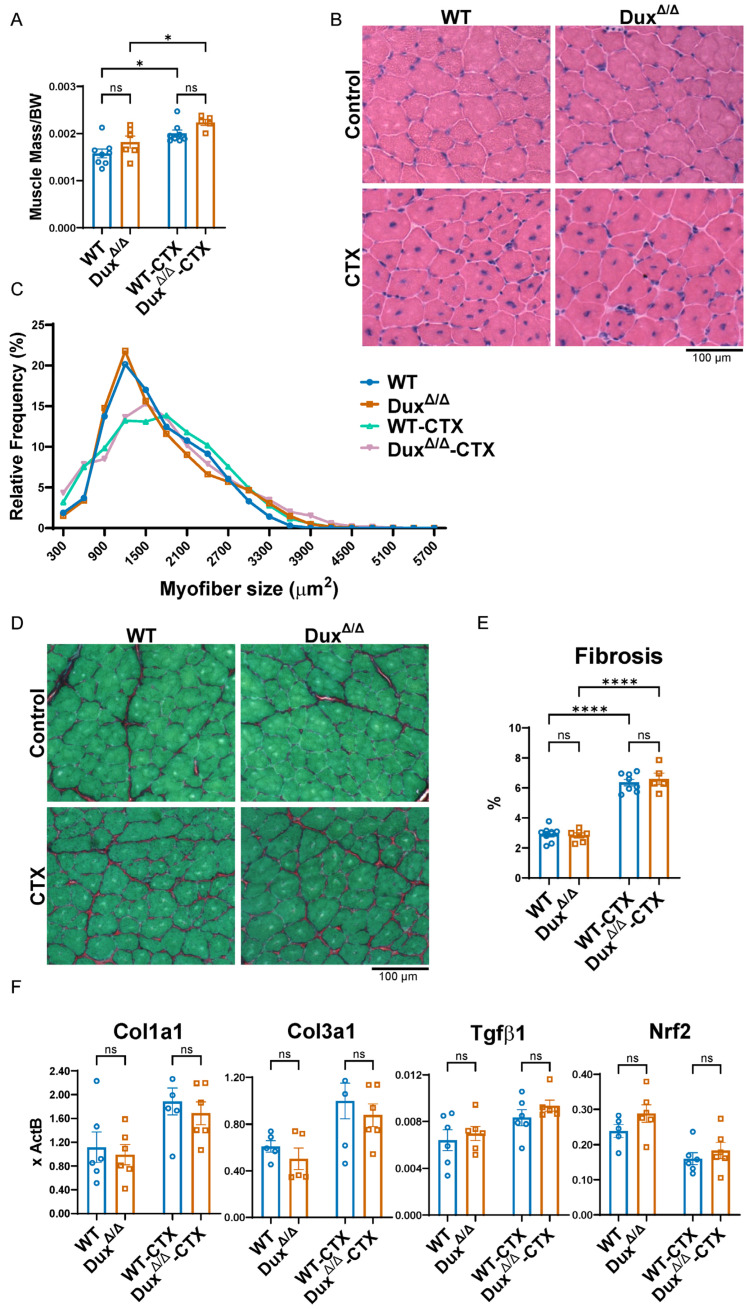
Loss of Dux does not affect muscle homeostasis. (**A**) Tibialis anterior (TA) muscle mass normalized to body weight in wild-type (WT) and Dux^Δ/Δ^ mice, either uninjured or two weeks post–cardiotoxin (CTX) injury (*n* = 5). (**B**) Representative H&E-stained images of TA muscle from WT and Dux^Δ/Δ^ mice. (**C**) Myofiber size distribution normalized to cross-sectional area (CSA) in uninjured and CTX-injured TA muscle. (**D**) Representative Sirius Red/Fast Green staining of TA muscle from WT and Dux^Δ/Δ^ mice. (**E**) Quantification of fibrosis in TA muscle based on staining shown in (**D**). (**F**) RT-qPCR analysis of fibrosis-related genes (*Col1a1*, *Col3a1*, *TGFβ*) and the oxidative stress marker *Nrf2*, normalized to *ActB*, in gastrocnemius muscle. Data are presented as mean ± SEM; (“ns” indicates not significant, * *p* < 0.05, **** *p* < 0.0001, one-way ANOVA, *n* = 5).

To investigate the potential role of Dux in skeletal muscle regeneration, we utilized our Dux^Δ/Δ^ mouse model to evaluate muscle morphology and regenerative capacity under both homeostatic and injured conditions [7]. While this experiment is not in an mdx background, if Dux impairs satellite cell function, as proposed by Sun et al. in the dystrophic context, its deletion would be expected to enhance regenerative outcomes even in a wild-type background. We examined two experimental conditions: comparison of intact TA muscle between wild-type (WT) C57BL/6 (B6) and Dux^Δ/Δ^ mice, and assessment of the regenerative response over a 4-week period following cardiotoxin (CTX) injury. We observed no significant difference in TA muscle mass between WT and Dux^Δ/Δ^ mice, either in the intact state or at 2 and 4 weeks post-injury (Figure 2A and Supplementary Figure S2A). Gross histological analysis showed normal muscle structure in both genotypes, with no discernible macroscopic differences following CTX injury (Figure 2B and Supplementary Figure S2B). Quantitative analysis of myofiber size and distribution revealed no significant differences between WT and Dux^Δ/Δ^ mice in either uninjured or regenerating muscle. Sirius Red/Fast Green staining indicated similar levels of fibrosis in both groups (Figure 2C,D and Supplementary Figure S2C,D). Additionally, RT-qPCR analysis of several fibrosis-associated genes demonstrated no differential expression (Figure 2F). Lastly, *Nrf2* transcript levels were also comparable between WT and Dux^Δ/Δ^ mice (Figure 2F).

These findings indicate that deletion of Dux does not affect skeletal muscle mass, fiber morphology, regenerative capacity, fibrosis, or *Nrf2* expression, suggesting that Dux does not play a functional role in muscle homeostasis or regeneration in WT mice.

## 4. Discussion

Initially motivated by doubts about the reliability of the presented data and further compelled to investigate after the editors ignored (twice) our concerns, we set out to rigorously examine the claims made in the published manuscript. We demonstrated that *Dux* is not expressed in murine skeletal muscle under normal, regenerative, or dystrophic (mdx) conditions by assessing *Dux* and its target gene expression in publicly available RNA-seq datasets [41]. Visualizing the origin of transcripts using RNA-seq data provides greater reliability than amplifying transcripts with primers that do not span introns, as was performed in this and previous studies [2,24]. Furthermore, we performed immunofluorescence using a validated antibody known to specifically detect Dux [34]. To confirm its specificity, we included appropriate positive and negative controls. We did not attempt to use the antibody employed in the original study, as there is no supporting evidence or reference demonstrating its specificity for mouse Dux. Based on these findings, we conclude that Dux is unlikely to play a significant role in muscle homeostasis or in the processes of muscle degeneration and regeneration, either in injury models or in the mdx context.

To further investigate the functional role of Dux in muscle regeneration, we employed our Dux^Δ/Δ^ mouse model, which lacks *Dux* expression and exhibits phenotypes consistent with its known role during early embryonic development [7]. Unlike the study by Sun et al., we did not evaluate the muscle phenotype in mdx/Dux^Δ/Δ^ mice, choosing instead to reduce the time and breeding costs associated with generating this genotype. Rather, we utilized the CTX injury model, a widely accepted approach for assessing satellite cell-mediated muscle regeneration [46,47]. In this context, we observed no differences in regenerative capacity between wild-type and Dux^Δ/Δ^ mice. These findings are consistent with previous studies that reported no obvious muscle phenotype in *Dux* knockout mice, further supporting the conclusion that the loss of *Dux* does not significantly impact muscle development or function [8,9].

Finally, analysis of RNA-seq data from muscle biopsies of DMD patients revealed no detectable *DUX4* transcripts or activation of its target genes [40,44], indicating that DUX4 is unlikely to contribute to the disease process. Consequently, these findings refute the scientific rationale for proposing anti-DUX4 therapies as a treatment strategy for DMD.

We do not intend to speculate on whether the findings reported by Sun et al. were fabricated [24]. However, without undertaking a full review of their paper, we wish to highlight several concerns that call into question the reliability of their findings. These include the lack of essential methodological details, such as the source and validation of key reagents like primers and antibodies, absence of appropriate experimental controls, failure to report the number of biological replicates, a limited description of the animal model used, including an initially erroneous explanation of its source, and the insufficient quality of histological data [24]. Additionally, certain findings, such as the report of greater than 20% fibrosis in the skeletal muscles of 8-week-old mdx mice, are inconsistent with previously published results, further undermining confidence in the validity of the study [48].

Beyond these scientific concerns, there are several broader red flags that raise questions about the study’s provenance [28]. These include signs often associated with publications originating from so-called “paper mills”: the research was reportedly conducted at a clinical institution in China by authors who appear to be clinicians without prior experience or publications in the field, the use of non-institutional email addresses for correspondence, and co-authors with no documented history of prior collaboration [29,30,31].

The proliferation of fraudulent scientific publications originating from “paper mills” has grown into a multimillion-dollar enterprise, operating at a scale that is still not fully understood [31,49]. Estimates suggest that approximately 2% of all submitted scientific manuscripts, and up to 3% in the fields of biology and medicine, are fraudulent [49], with a significant portion originating from countries such as China, Russia, and India [50]. This trend is fueled in part by systemic pressures within academia, where “publish or perish” cultures and promotion criteria often prioritize the quantity of publications over the quality and impact of the research itself. In some countries, these pressures are further intensified by institutional mandates requiring clinicians, many of whom have minimal direct engagement in research, to publish scientific articles as a prerequisite for career advancement [50,51]. Compounding the issue is the financial incentive for publishers, as an increased volume of publications directly translates into greater revenue. This environment has paved the way for questionable, and at times entirely fraudulent studies to reach publication, leading to a surge in the number of retractions, which exceeded 10,000 in 2023 alone [52].

This growing trend poses a significant threat to the integrity and effectiveness of scientific research. Fraudulent publications not only mislead researchers and distort the scientific record but also undermine public trust in science. The ramifications are particularly serious in fields of high clinical relevance, such as drug and vaccine development, where misinformation or flawed data may delay vital breakthroughs or lead to the misallocation of valuable resources [53].

In summary, we aimed to highlight the relevance and risks posed by fraudulent publications, especially in areas with direct implications for patient care, such as DMD. We also underscore the responsibility of editors and journals to uphold scientific standards. In this case, our concerns regarding a questionable study were dismissed without thorough consideration, representing a missed opportunity to protect the integrity of the field. We hope that by drawing attention to these issues, we can encourage more rigorous editorial oversight and foster a collective effort to preserve scientific credibility.

## Data Availability

The original contributions presented in this study are included in the article; further inquiries can be directed to the corresponding author.

## Supplementary Materials

The following supporting information can be downloaded at: https://www.mdpi.com/article/10.3390/cells14100695/s1, Figure S1: DUX4 is not expressed in DMD muscle biopsies; Figure S2: Muscle assessment at 4 weeks post-injury.
