# Supplementary material for: Dux Is Dispensable for Skeletal Muscle Regeneration: A Study Inspired by a “Red Flagged” Publication and Editorial Oversight
Source: Cells. 2025 May 12;14(10):695. doi: 10.3390/cells14100695 (PMC12109671; doi:10.3390/cells14100695)
Supplement: Supplementary file 1 [file cells-14-00695-s001.zip › cells-3622826-supplementary.pdf]

cells

MDPI

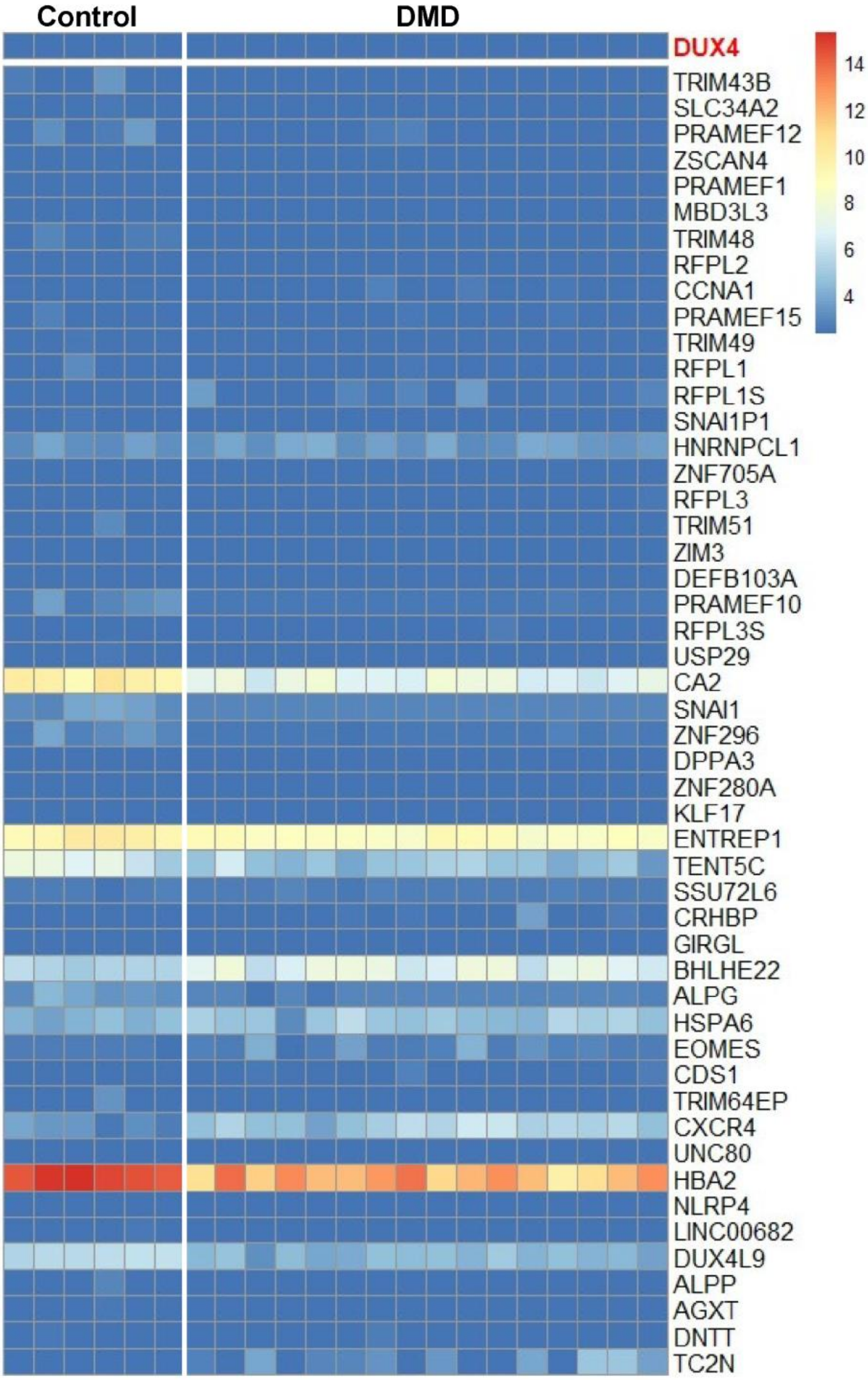

Supplementary Figure S1. DUX4 is not expressed in DMD muscle biopsies. Heatmap showing DUX4 and DUX4 target gene expression in muscle biopsy samples from Duchenne muscular dystrophy (DMD) patients (*43*).

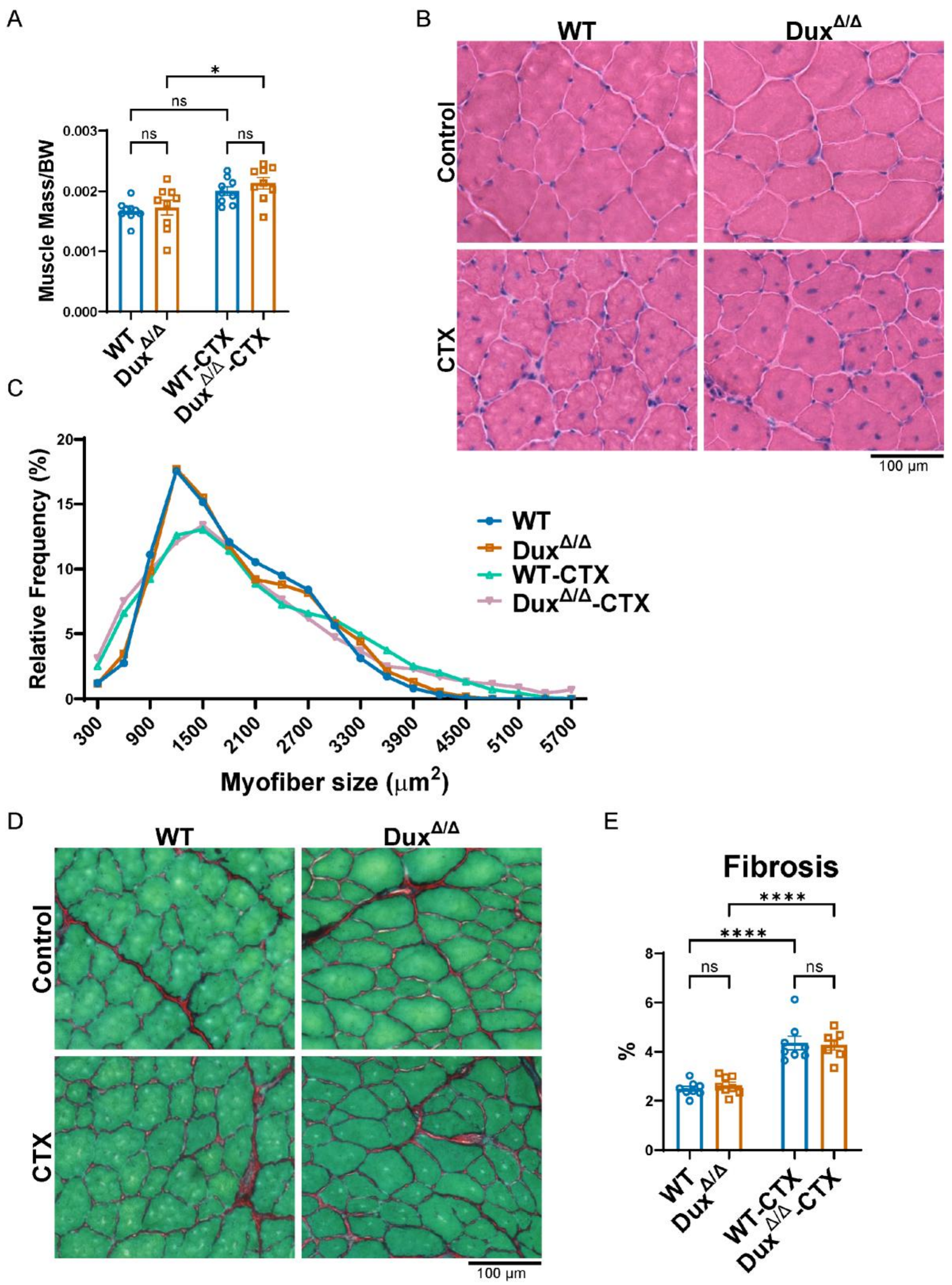

Supplementary Figure S2. Muscle assessment at 4 weeks post-injury. (A) Tibialis anterior (TA) muscle mass normalized to body weight in wild-type (WT) and Dux$^{\Delta/\Delta}$ mice, either uninjured or two weeks post–cardiotoxin (CTX) injury (n = 9). (B) Representative H&E-stained images of TA muscle from WT and Dux$^{\Delta/\Delta}$ mice. (C) Myofiber size distribution normalized to cross-sectional area (CSA) in uninjured and CTX-injured TA muscle. (D) Representative Sirius Red/Fast Green staining of TA muscle from WT and Dux$^{\Delta/\Delta}$ mice. (E) Quantification of fibrosis in TA muscle based on staining shown in (D). Data are presented as mean ± SEM; (p*> 0.05, p****> 0.0001, one-way ANOVA, n=9).
